# Factors associated with history of drug use among female sex workers (FSW) in a high HIV prevalence state of India

**DOI:** 10.1186/1471-2458-12-273

**Published:** 2012-04-05

**Authors:** Gajendra Kumar Medhi, Jagadish Mahanta, Michelle Kermode, Ramesh S Paranjape, Rajatashuvra Adhikary, Sanjib Kumar Phukan, P Ngully

**Affiliations:** 1 Regional Medical Research Centre (RMRC), N.E. Region (ICMR), Dibrugarh, 786001, Assam, India; 2 Technical Director, Northeast India Knowledge Network, Nossal Institute for Global Health, University of Melbourne, Carlton, Victoria, 3010, Australia; 3 National AIDS Research Institute (NARI), Plot No. 73, Block G, MIDC Complex, Bhosari, Pune, 411026, India; 4 FHI 360, 1825 Connecticut Avenue, Washington, DC, 20009, USA; 5 Kripa Foundations, Kohima, 797001, Nagaland, India; 6 Regional Medical Research Centre, NE Region, Indian Council of Medical Research, Dibrugarh, 786001, Assam, India

**Keywords:** FSW, Drug Use, HIV, STIs, Condom Use

## Abstract

**Background:**

The intersection between illicit drug use and female commercial sex work has been identified as an important factor responsible for rising HIV prevalence among female sex workers (FSW) in several northeastern states of India. But, little is know about the factors associated with the use of drugs among FSWs in this region. The objective of the paper was to describe the factors associated with history of drug use among FSWs in Dimapur, an important commercial hub of Nagaland, which is a high HIV prevalence state of India.

**Methods:**

FSWs were recruited using respondent driven sampling (RDS), and were interviewed to collect data on socio-demographic characteristics and HIV risk behaviours. Biological samples were tested for HIV, syphilis gonorrhea and Chlamydia. Logistic regression analysis was performed to identify factors associated with drug use.

**Results:**

Among the 426 FSWs in the study, about 25% (n = 107) reported having ever used illicit drugs. Among 107 illicit drug users, 83 (77.6%) were non-injecting and 24 (22.4%) were injecting drug users. Drug-using FSWs were significantly more likely to test positive for one or more STIs (59% vs. 33.5%), active syphilis (27.1% vs. 11.4%) and Chlamydia infection (30% vs. 19.9%) compared to their non-drug using peers. Drug-using FSWs were also significantly more likely to be currently married, widowed or separated compared with non-drug-using FSWs. In multiple logistic regression analysis, being an alcohol user, being married, having a larger volume of clients, and having sexual partners who have ever used or shared injecting drugs were found to be independently associated with illicit drug use.

**Conclusions:**

Drug-using FSWs were more vulnerable to STIs including HIV compared to their non-drug using peers. Several important factors associated with being an FSW who uses drugs were identified in this study and this knowledge can be used to plan more effectively targeted harm reduction strategies and programs.

## Background

The intersection between illicit drug use and female commercial sex work has been recognized for many years [1]. Drug use among female sex workers (FSWs) is an important public health concern because of the link with HIV transmission. Injecting drug use is a major route of HIV transmission and other blood-borne infections among FSWs in a variety of settings [1-5]. Several studies have also documented substantially higher prevalence of HIV and other infections among non-injecting drug users compared to non-drug users [3,6-10]. Several plausible explanations account for the higher HIV prevalence among non-injecting drug users compared to the general population [8].

The overlap between illicit drug use and female sex work has been identified as an important factor responsible for rising HIV prevalence among FSWs in several northeastern states of India [4]. The state of Nagaland borders Myanmar and is one of the highest HIV prevalence states in India – in 2009 the prevalence among the general population was 0.8% [11]. The HIV epidemic in Nagaland has historically been attributed to the high prevalence of injecting drug use (mostly with a propoxyphene-based pharmaceutical Spasmoproxyvon, and heroin), but the importance of sexual transmission has been increasingly recognized in recent years. Surveillance reports indicate that HIV prevalence among injecting drug users (IDUs) in Nagaland has reduced from 8.4% in 2003 to 1.9% in 2007, whereas the prevalence among FSWs went up sharply from 4.4% in 2003 to 16.5% in 2006, and still remains above 10% [12,13]. Drug involvement is reported to be the main factor for such sudden increase of HIV among them [14]. A previous study of FSWs in Nagaland (n = 220) found that about 15% had entered sex work to support their drug use and that, at the time of entry into sex work, 60% were regularly using alcohol or other drugs, 17% were using heroin and 6% had ever injected drugs [15,16]. To date, very little is known about the characteristics of FSWs who use illicit drugs in this region. It is important to identify the factors associated with this highly vulnerable sub-group of FSWs so that effective HIV prevention strategies can be appropriately targeted. The present study was conducted in Dimapur, which is the commercial capital of Nagaland, and the main hub for commercial sex work. The specific objective of this paper is to identify factors associated with illicit drug use among FSWs in Nagaland in order to more effectively target this high risk group with HIV prevention interventions.

## Methods

### Study design

This cross–sectional study among FSWs was conducted in the Dimapur district of Nagaland from February to April 2006 as a part of large multi-centre study known as the Integrated Biological and Behavioural Assessment (IBBA). A detailed summary of the IBBA objectives, sampling methods used, and questionnaire are described elsewhere [17,18].

### Sampling design

Respondent driven sampling (RDS) was adopted to recruit study participants. RDS is a variant of chain-referral sampling which has been proved to be feasible and successful to recruit hidden populations such as injecting drug users (IDU), FSWs and men who have sex with men (MSM) in a more representative manner [19-21]. RDS method allows calculating asymptomatic unbiased estimates of population parameters extenuating the biases of chain referral sampling [22] using RDSAT (RDS analysis tool). In RDS, sampling begins with few non-randomly selected initial recruits (called ‘seed’) from the target population who meet the eligibility criteria. These seeds then start the chain referral by recruiting fixed numbers of eligible peers from their personal network who, in turn, recruit other peers for the study. This recruitment process continues until the target sample size is attained [19]. The target sample size for the study was calculated as 400 [14,18]. In this study, we recruited ten purposively selected seeds to initiate recruitment process. Then, all the seeds were given three uniquely coded coupons to recruit three eligible peers from their personal networks. This recruitment process continued till the target sample size was achieved in the study. One seed could not produce any recruits; while other two seeds propagated only upto 2nd waves. Number of waves for other seven seeds ranged from 6 to 11. Only about 8 weeks was required to recruit the required samples in the study. More detailed description of sampling design adopted to recruit participants in this study has been already described elsewhere [14,17,18].

### Data collection and variables

The definition of an FSW was a female, aged 18 years or older, who had sex with men in exchange for cash or kind at least once within the past one month. Anonymous face to face interviews were conducted by trained female interviewers who collected data using a structured questionnaire after obtaining written informed consent from the eligible participants. The dependent variable was lifetime use of illicit drugs (injecting and/or consuming illicit drugs) among FSWs. Respondents were asked to report if they had ever consumed or ever injected any illicit drugs (e.g., spasmoproxivon, heroin, marijuana, and methamphetamine etc.). Other variables of interest were socio-demographic characteristics, patterns of sex work, sexual risk behaviors, and knowledge about HIV. The socio-demographic variables included respondents’ age, educational status, and marital status. Variables related to sex work and sexual practices included consistent condom use with sex partners (regular, occasional clients and regular non-paid partners), volume of clients per week, types of clients, venues for soliciting and having sex with clients, age at first sexual experience. Consistent condom use was defined as the every time condom use during a sexual act.

The participants were also asked to provide blood and urine samples. Blood samples were tested for HIV and syphilis. Urine samples were tested for *Neisseria gonorrhoeae (NG)* and *Chlamydia trachomatis (CT)*. Serum samples were tested for HIV by Microelisa (J. Mitra and Company, India), and positive tests were confirmed by Genedia HIV 1/2 ELISA 3.0 (Green Cross Life Science Corporation, South Korea). Serum samples were also tested for syphilis by rapid plasma reagin (RPR) test and confirmed by *Treponema pallidum* haemagglutination assay (TPHA). Urine samples were tested with nucleic acid amplification assay (Gen-Probe Aptima) for the detection of *Neisseria gonorrhoeae* and *Chlamydia trachomatis*. The methods and procedures adopted for testing the biological samples have been described elsewhere in details [18]. Having a sexually transmitted infection (STI) was defined as a positive test result for any of the gonorrhea, chlamydia and/or syphilis.

### Statistical analysis

We compared the characteristics of drug-using FSWs and non-drug-using FSWs using Pearson’s Chi-square test. RDS-weighted univariate and multiple logistic regression analyses was used to estimate crude and adjusted odds ratios (OR, 95% confidence interval) using SPSS software to identify the factors associated with illicit drug use among FSWs. Only variables that were found to be significantly associated with drug use in univariate analysis at 5% level were included in the multiple logistic regression model to identify the factors independently associated with drug use. The individualized weights generated for the dependent variable (i.e. ever drug use) using RDS Analysis Tool version 5.6 [23] were applied to the logistic regression analysis using SPSS in order to adjust for the RDS sampling process [23].

### Ethical approval

The Health Ministry Screening Committee of the Government of India and ethical review bodies of the participating institutions (Family Health International, Regional Medical Research Center, and National AIDS Research Institute) granted ethical approval for the study.

## Results

Of the 426 FSWs who took part in the study, 107 (25%) reported having ever used illicit drugs for non-medical reasons. Of the 107 illicit drug users, 83 were exclusively oral drug users, 2 were exclusively injecting drug users, and 22 were both oral and injecting drug users. Three individuals were excluded from the further analysis due to their non-discloser about drug use behaviour.

Table 1 displays the characteristics of the sample of FSWs disaggregated by drug-using status. Drug-using-FSWs were older compared with non-drug-using FSWs, but the groups did not differ significantly in terms of their educational status or age of sexual debut. A higher proportion of drug-using FSWs was widowed (15% vs. 8.9%), separated (24.3% vs. 19.6%) and married (44.9% vs. 38.3%).

**Table 1:** Characteristics of FSW participants disaggregated by drug using status

**Characteristics**	**Ever used drugs (N = 107) n (%)**	**Never used drugs (N = 316) n (%)**	***p*-value**
**Current age (years)**			
18–19	13 (12.1)	74 (23.4)	0.018
20–24	27 (25.2)	88 (27.8)	
25+	67 (62.6)	154 (48.7)	
*Mean age*	*26*	*25*	
*Median age*	*25*	*24*	
**Marital status**			
Married	48 (44.9)	121 (38.3)	0.005
Divorced/separated	26 (24.3)	62 (19.6)	
Widowed	16 (15.0)	28 (8.9)	
Unmarried	17 (15.9)	105 (33.2)	
**Educational status**			
Illiterate	52 (48.6)	139 (44.0)	0.666
Upto 10^th^ standard	48 (44.9)	151 (47.8)	
> 10^th^ standard	7 (6.5)	26 (8.2)	
**Consumed alcohol (past month)**			
Everyday	34 (31.8)	40 (12.6)	<0.001
At least once a week	48 (44.9)	121 (38.3)	
Never/< once a week	25 (23.4)	155 (49.1)	
Have sexual partners who have used or shared injecting drugs (ever) *			
No	42 (39.3)	239 (75.6)	<0.001
Yes	63 (58.9)	70 (22.1)	
Age at first sex*			
<=15 years	49 (45.8)	131 (41.5)	0.447
> 16 years	58 (54.2)	184 (58.2)	
**Place of sex work#**			
Hotel/brothel	77 (72)	177 (56)	0.014
Public place	6 (5.6)	24 (7.6)	
Home	24 (22.4)	115 (36.4)	
Duration of sex work*			
< 2 years	12 (11.2)	59 (18.7)	0.019
2–4 years	39 (36.4)	144 (45.7)	
5–9 years	37 (34.6)	72 (22.9)	
> = 10 years	19 (17.8)	40 (12.7)	
*Mean duration (years)*	*5.91*	*4.83*	
Client volume per week*			
0–4	31 (29.0)	156 (49.4)	0.001
5–9	49 (45.8)	108 (34.2)	
> = 10	27 (25.2)	51 (16.1)	
*Mean no. clients/week*	*7.72*	*5.72*	
Condom use with occasional clients*			
Sometimes/never	56 (52.3)	221 (70.0)	0.002
Most of the time	24 (22.4)	49 (15.5)	
Every time	27 (25.2)	42 (13.3)	
Condom use with regular clients*			
Sometimes/never	73 (68.2)	248 (78.5)	0.034
Most of the time	19 (17.8)	39 (12.3)	
Every time	15 (14.0)	23 (7.3)	
**Condom use with main regular partners**			
Sometimes/never	98 (91.6)	283 (89.6)	0.815
Most of the time	5 (4.7)	17 (5.4)	
Every time	4 (3.7)	16 (5.0)	
Ever heard of HIV*			
No	6 (5.6)	46 (14.6)	0.014
Yes	101 (94.4)	269 (85.1)	
**CT**			
Negative	76 (70.0)	253 (80.1)	0.052
Positive	31 (30.0)	63 (19.9)	
**NG**			
Negative	100 (93.4)	305 (96.5)	0.175
Positive	7 (6.54)	11 (3.5)	
**Active syphilis**			
No	78 (72.9)	280 (88.6)	
Yes	29 (27.1)	36 (11.4)	<0.001

N.B: ‘#’Hotel/brothel = bar, night club, brothel, dhaba, hotel and lodge.

Public place = public place, vehicle, others.

Home = home (client/FSWs) and rented room.

‘*’Data with missing cases.

Drug-using FSWs were more likely to have consumed alcohol at least once weekly in the past month compared to non-drug-using FSWs, and they reported more sexual relationships with IDU clients who shared needles compared to non-drug-using FSWs (58.9% vs. 22.1%). The average duration of sex work was longer for drug-usingFSWs compared to non-drug-using FSWs (*6 years vs 4.8 years*), and they reported more clients in the last working week (*7.7 clients vs 5.7 clients*). A higher proportion of drug-using FSWs served their clients at lodges/hotels (72% vs. 56%), and they were more likely to report using condoms for every sexual act with both regular (14% vs. 7.3%) and occasional clients (25.2% vs. 13.3%). The proportion of FSWs who used condoms consistently with their main regular sex partners (husband/boy-friends) was very low for both groups (*5% and 3.7%*) (Table 1).

Compared to non-drug-using FSWs, drug-using FSWs were significantly more likely to test positive for HIV, syphilis, and Chlamydia infection. Overall, at least one STI (syphilis, Chlamydia, and/or gonorrhea) was detected among 59% (n = 63) of drug-using FSWs compared to 33.5% (n = 106) among non-drug-using FSWs. A higher proportion of drug-using FSWs had HIV (25.2% vs. 9.5%), syphilis (27.1% vs. 11.4%), and Chlamydia (30% vs. 19.9%). Drug-using FSWs were more likely to have heard about HIV compared to their non-drug-using peers (94.4% vs. 85.1%).

The results of univariate and multiple logistic regression analysis are shown in the Table 2. In the univariate analysis, compared with non-drug-using FSWs, drug-using FSWs were more likely to: be older (>25 years of age); be widowed/separated or currently married; consume alcohol (daily/at least once a week during last month); have sexual partners who have ever used or shared injecting drugs; have sex with clients at lodges/hotels; have a longer duration in sex work (>4 years); have served 5 or more clients in the last working week; use condoms consistently while having sex with regular clients; use condoms consistently or most of the time while having sex with occasional clients; have heard about HIV/AIDS; and have active syphilis. Although statistically non-significant, the odds of being a drug-using FSW were about 2-times higher for those who tested positive for NG and 1.6 times higher for those who tested positive for CT.

**Table 2:** Factors associated with ever drug use in univariate (crude OR) and multiple (adjusted OR) logistic regression analysis

**Characteristic**	**Crude OR**	**95% CI**	**Adjusted OR**	**95% CI**
**Current age (years)**				
18–19	Reference		Reference	
20–24	1.75	0.80–3.84	1.56	0.57–4.27
25+	2.48*	1.22–5.02	1.20	0.43–3.38
**Marital status**				
Married	2.45**	1.27–4.74	2.33*	1.01–5.40
Divorced/separated	2.59*	1.24–5.42	2.55	0.88–5.80
Widowed	3.53**	1.50–8.28	2.58	0.84–7.95
Unmarried	Reference		Reference	
**Educational status**				
Illiterate	1.39	0.533–3.62		
Up to 10^th^ standard	1.18	0.45–3.09		
> 10^th^ standard	Reference			
**Consumed alcohol (past month)**				
Everyday	5.27**	2.72–10.20	5.15**	2.27–11.66
At least once a week	2.46**	1.38–4.39	3.01**	1.45–6.23
Never/< once a week	Reference		Reference	
Have sexual partners who have used or shared injecting drugs (ever) *				
No	Reference		Reference	
Yes	5.12**	3.13–8.45	4.91**	2.69–8.92
**Place of sex work**				
Hotel/brothel	2.09**	1.20–3.62	1.20	0.59–2.42
Public place	1.20	0.41–3.50	1.40	0.34–5.76
Home	Reference		Reference	
**Age at first sex**				
<=15 years	1.19	0.74–1.90		
> 16 years	Reference			
**Duration of sex work**				
< 2 years	Reference		Reference	
2–4 years	1.33	0.62–2.88	0.73	0.28–1.90
5–9 years	2.53*	1.15–5.57	1.42	0.48–4.18
> = 10 years	2.34*			0.96–5.66	1.92	0.55–6.66
**Client volume per week**						
0–4	Reference		Reference			
5–9	2.28**	1.32–3.95	2.59**	1.30-5.13		
> = 10	2.66**	1.40-5.08	2.87**	1.30–6.34		
**Condom use with occasional clients**						
Sometimes/never	Reference		Reference			
Most of the time	1.93*	1.06–3.54	1.25	0.49–3.13		
Every time	2.54**	1.40–4.61	1.98	0.69–5.68		
**Condom use with regular clients**						
Sometimes/never	Reference		Reference			
Most of the time	1.66	0.87–3.15	1.69	0.62–4.61		
Every time	2.22*	1.06–4.64	0.89	0.24–3.25		
**Condom use with main regular partners**						
Sometimes/never	Reference					
Most of the time	1.39	0.42–4.61				
Every time	1.16	0.24–5.77				
**Ever heard of HIV**						
No	Reference		Reference			
Yes	2.88*	1.11–7.48	2.74	0.91–8.22		
**CT**						
Negative	Reference					
Positive	1.64	0.96–2.79				
**NG**						
Negative	Reference					
Positive	1.94	0.70–5.41				
**Active syphilis**						
No	Reference		Reference			
Yes	2.98**	1.67-5.33	1.79	0.85–3.79		

'^*'^ refers *p*-value < 0.05 and '^'**^' for *p*-value < 0.01.

Additional analysis indicates that the factors significantly associated with illicit drug use (oral and/or injecting drug use) remained fairly constant even when the injectors were removed from the group, with the exception of age, duration of sex work and having ever heard of HIV (Table not shown).

In the multiple regression analysis, after controlling for all the significant factors in univariate analysis, daily alcohol use (OR = 5.15, CI: 2.27–11.66), alcohol use at least once a week (OR = 3.01, CI: 1.45–6.23), being widowed (OR = 2.58, CI: 0.84–7.95), being separated (OR = 2.55, CI: 0.88–5.8), serving 5–9 (OR = 2.59, CI: 1.30–5.13) or more than 9 clients (OR = 2.87, CI: 1.30–6.34) in the last working week, and having sexual partners who have ever used or shared injecting drugs (OR = 4.91, CI: 2.69–8.92) were all found to be factors significantly associated with a history of ever having used illicit drugs.

## Discussion

This study found that about a quarter of FSWs in Dimapur district of Nagaland reported having ever used illicit drugs. The drug-using FSWs substantially differed from their non-drug-using peers in terms of their demographic profile, sexual risk profile and burden of sexually transmitted diseases.

Younger FSWs were less likely to have ever used drugs, which may reflect a true declining trend of drug use. It is also possible that those who remain in sex work for longer are more likely to have been exposed to drugs, and to have begun to use them as a means of coping with the challenges inherent in being an FSW in this context. An earlier study of FSWs in Dimapur noted an increase in the proportion of FSWs regularly using alcohol and other drugs after the commencement of sex work [16]. In terms of marital status, we found that those FSWs who were or had been married were more likely to report drug use compared to those who had never married. Furthermore, among the married FSWs those who were widowed were more vulnerable to drug use. The probability of using drugs among the married group remained elevated even in the multivariate model indicating an independent relationship. The drug use of husbands’ seems to play an important role in the development of drug use among married women in India [24]. A higher rate of drug use among widows may be related to the influence of their husband’s drug use, as many young widows in Nagaland are in this situation due to the drug-related death of their husbands [25]. Drug use by one or both marital partners contributes to family disharmony, and can lead to separation or divorce [24]. These drug-using women may be forced into sex work in order to sustain their livelihood and their drug use [4,15]. In this study, drug-using FSWs and non-drug-using FSWs did not differ from one another in terms of their educational status. Our results are contrary to previous reports from this region that indicated a strong link between illiteracy and substance use for women [26-29].

This study also found that alcohol use among drug-using FSWs was more common compared with non-drug-using FSWs. This finding is consistent with previous reports, which showed that co-use of alcohol and illicit drugs was very common among drug users in northeast India, including female drug users [4,24,30]. Consuming alcoholic drinks at least once weekly was independently associated with having ever used drugs among FSWs in this study. Panda et al. (2006) reported that 98% female drug users consumed alcohol prior to use of illicit drug in Manipur [4]. This is certainly a cause for concern because concomitant use of drugs and alcohol are synergistic risk factors for transmission of HIV and other STIs [31]. Probably, alcohol use can be used as a behavioural marker for identifying suspected illicit drug users as illicit drug use is a more stigmatized and socially unacceptable behaviour particularly among women in comparison to alcohol use. FSWs may not be hesitant to disclose their habit of consuming culturally acceptable home brewed alcohol.

Studies in this region indicate that many female drug users resort to sex work to obtain drugs from their sexual partners or to earn money for purchasing drugs [4,15,24]. The drug-using FSWs in our study were more likely to report sexual interactions with clients who had ever injected or shared injecting drugs, and they also reported a higher volume of clients compared with non-drug-using FSWs. Volume of clients was found to be independently associated with being a drug-using FSW. Overall, our data indicate that drug-using FSWs are more likely to be exposed to HIV and other STIs due to their higher volume of clients and more sexual interactions with male drug users.

Contrary to previous reports, in our study drug-using FSWs compared to non-drug-using FSWs were more likely to report using condoms consistently or most of the time with both regular and occasional clients [9]. But, such results should be interpreted with caution since studies have shown that sex work conducted under the influence of drugs is associated with lower rates of condom use in other settings [32-34]. One possible explanation for the higher rate of condom use among drug-using FSWs in the context of Nagaland may be their greater awareness about the importance of condom use, since drug users have been a major target group for HIV prevention programmes in this region for a long time. In fact, we also found that prior knowledge of HIV was strongly associated with being a drug-using FSW. The venue for having sex with clients was significantly associated with having ever used illicit drugs in the univariate analysis. We found that drug-using FSWs were more likely to operate from lodges/hotels and least likely to be street-based. Probably lodges/hotels were used clandestinely for the dual purposes of drug use and sex by FSWs.

In our previous report from this study, we showed that both injecting and oral drug use were significantly associated with HIV among FSWs in Dimapur [14]. Prevalence of HIV was almost three times higher among drug-using FSWs compared to non-drug using group [14]. This analysis throws further light on the relationship between drug use and other STIs among FSWs. We could not establish a significant multivariate association between drug use and individual STIs in this study, but prevalences of individual STIs were generally higher among drug-using FSWs. Similarly, the overall prevalence of one or more STIs was almost two times higher among drug-using FSWs compared to non-drug-using FSWs (59% vs. 33.5%) indicating their greater vulnerability to STIs. Even after exclusion of the IDUs from the drug-using group, prevalence of one or more STIs was significantly higher among life time illicit drug users compared with non-drug-using FSWs (results not shown) highlighting the importance of illicit drug consumption in the epidemiology of HIV and other STIs among FSWs in this region. These findings suggest that the drug-using FSWs need to be targeted by the prevention programs with greater urgency to make the HIV and STI prevention programs more successful.

This study has certain limitations. Firstly, RDS was used to recruit FSWs in the study because random selection of the target population was not a possibility according to our pre-survey assessment. However, RDS can be considered as the best sampling option to obtain better representative samples of a target population where random sampling is not feasible. The statistical theory upon which RDS is based suggests that if peer recruitment proceeds through a sufficiently large number of waves, the composition of the sample will stabilize, becoming independent of the seeds from which recruitment began, and thereby overcoming any bias the nonrandom choice of seeds may have introduced [35]. This stable sample composition is termed the “equilibrium”. Therefore, it is after the point of equilibrium that the sample becomes representative of the study population [36]. In this study, we could achieve equilibrium for most of the key characteristics after 5^th^–6^th^ waves. Secondly, we relied on self-reported data regarding sensitive personal information such as use of illicit drugs and sexual behaviours, hence the results are subject to social acceptability bias. To improve the veracity of the self-reported data, participants were assured of anonymity, and told that there would be no adverse consequences for disclosing this information to the interviewers. Further, it is difficult from a cross-sectional study to establish temporal relationships between dependent and independent variables. Lastly, only data regarding ever drug use was collected in this study. Therefore, we are unable to describe the extent and pattern of current drug use among FSWs in Dimapur, and the relationship between these variables, their sexual risk behaviours, and infection with HIV and STIs.

## Conclusions

This is the first ever study from northeast India to demonstrate that predominantly non-injecting drug-using-FSWs were disproportionately more vulnerable to HIV and other STIs. The impact of HIV prevention efforts is likely to be enhanced if this more vulnerable sub-group of FSWs were effectively targeted. Several factors such as alcohol use, marital status, larger volume of clients, and sexual partners’ drug injecting status were identified as having association with drug use among FSWs. The findings of this report will be helpful in planning future harm reduction strategies among the FSWs in the region.

## Competing interests

The authors declare that they have no competing interests.

## Authors’ contributions

JM, RSP, RA contributed to the study design, review of the manuscript. GKM is responsible for the concept of the manuscript, drafting of the paper, data acquisition and data analysis. PN contributed to the data acquisition and interpretation of data. SKP was involved in the data management and analysis. MK contributed to the data interpretation and review of the manuscript. All authors read and approved the final manuscript.

## Pre-publication history

The pre-publication history for this paper can be accessed here:

http://www.biomedcentral.com/1471-2458/12/273/prepub
